# Porous Cu-MOF nanostructures with anticancer properties prepared by a controllable ultrasound-assisted reverse micelle synthesis of Cu-MOF

**DOI:** 10.1186/s13065-022-00804-2

**Published:** 2022-03-05

**Authors:** Reza Akhavan-Sigari, Malihe Zeraati, Mohammadreza Moghaddam-Manesh, Parya Kazemzadeh, Sara Hosseinzadegan, Narendra Pal Singh Chauhan, Ghasem Sargazi

**Affiliations:** 1 Department of Neurosurgery, University of Nebraska Medical Center, Tuebingen, Germany; 2 Department of Materials Engineering, Shahid Bahonar University of Kerman, 761694111 Kerman, Iran; 3 Petrochemistry and Polymer Research Group, Chemistry and Petrochemistry Research Center, Standard Research Institute, Tehran, Iran; 4 Department of Chemistry, Lorestan University, Khorramabad, Iran; 5 Department of Chemistry, Faculty of Science, University of Sistan and Baluchestan, Zahedan, Iran; 6 Department of Chemistry, Faculty of Science, Bhupal Nobles’ University, Udaipur, Rajasthan India; 7 Noncommunicable Diseases Research Center, Bam University of Medical Sciences, Bam, Iran

**Keywords:** Cu-MOF, Characterization, Cell viability, Anticancer

## Abstract

The ultrasonic assisted reverse micelle method (UARM) was used to synthesize Cu-MOF from Cu(NO_3_)_2_·3H_2_O and 2,6-pyridine dicarboxylic acid in a 1:1 molar proportion. It has been characterized using FT-IR, XRD, nitrogen adsorption analysis, SEM and TEM–EDX. The morphology of Cu-MOFs was spherical, with an average particle size distribution of less than 100 nm. Using BET analysis, the surface area of Cu-MOF was found to be 284.94 m^2^/g. The porous morphology of Cu-MOF was also suggested by SEM and TEM analyses. It has anticancer properties against MCF-7 breast cancer cells. Cytotoxicity testing was performed on MCF-7 breast cancer cells using the MTT cell viability assay, and cell proliferation and viability were found to be approximately 24% higher than the control.

## Introduction

Metal organic frameworks (MOFs) are a type of porous material made up of strong bonds between metal ions and organic linkers [1, 2]. MOFs with a careful constituent selection can have a very high surface area, a large pore volume, and excellent chemical stability. By careful selection of constituents, MOFs can exhibit very high surface area, large pore volume, and excellent chemical stability [3]. Research on synthesis, structures and properties of various MOFs has shown that they are promising materials for many applications, such as energy storage, gas storage, heterogeneous catalysis and sensing [4]. Apart from direct use, MOFs have also been used as support substrates for nanomaterials or as sacrificial templates/precursors for preparation of various functional nanostructures. Ding and co-workers have reviewed MOFs based nanozymes for the treatment of cancer [5–7].

Unfortunately, cancer is one of the leading causes of death for millions of people worldwide. Cancer biology has advanced significantly in the last decade, but cancer mortality remains high [8, 9]. Traditional drugs for cancer treatment have limitations such as poor pharmacokinetics, poor biological distribution, and adverse side effects [10]. Chemotherapy is the most commonly used cancer treatment, but it has several drawbacks, the most significant of which are low therapeutic efficiency in the treatment process and side effects on normal cells [8]. Since the 1970s, drug release control in drug delivery has been expanding [10]. Drug delivery systems based on nanoparticles are one of the newer methods in drug delivery systems. Drug delivery systems based on nanoparticles can avoid these issues while also increasing efficiency through targeted drug delivery, controlled release, and drug degradation protection. MOFs, d layered double hydroxides (LDHs), graphene oxide (GO), and magnetite are some examples of popular drug delivery systems that use nanoparticles today [8]. Some cases that have been investigated in the use of MOFs as drug delivery systems include excellent surface, thermal and chemical stability, high pore volumes, regular porosity and easy operation. [8, 10] Linxin et al. have prepared Zn_2_(EBNB)_2_(BPY)_2_.2H_2_O having drug delivery applications [11]. Numerous biological reports have been reported from organic metal frameworks, including inhibition of human glioma cell growth [12], anticancer activity [13–15], and antimicrobial properties [16–18]. One of the MOFs with high biological properties is Cu-MOF [19–21]. It is worth noting that the true nature of the active sites in many MOFs containing metal ions is saturated with the coordination of organic ligands [22]. Cu-MOF has superior antibacterial and anticancer properties [23, 24]. The bactericidal mechanism of Cu-MOF is due to the diffusion of Cu^2+^ ions. In addition, the negative charges of lipoproteins are absorbed into the cell wall, they enter the cell and damage the cell wall, alter the function of the enzymes of the cell wall, or create cell wall holes [25].

In this paper, we describe the synthesis of Cu-MOFs using the UARM method, followed by FT-IR, XRD, nitrogen adsorption, SEM, and TEM analyses. Its anticancer activity against MCF-7 breast cancer cells is being studied as well.

## Experimental section

### Materials

Cu (NO_3_)_2_·3H_2_O and 2, 6 pyridine dicarboxylic acid were purchased from Merck. C_12_H_25_NaSO_4_ was purchased from Sigma-Aldrich. Double distilled water (DDW) was used in each experiment.

### Method

Cu(NO_3_)_2_·3H_2_O (Merck, 98%) and 2, 6 pyridine dicarboxylic acid (Merck, 99%) are mixed with 1:1 mmol dissolved in 25 mL of DDW during the preparation of the samples using the ultrasonic assisted reverse micelle method. The resulting solution was added to a mixture of 0.077 mmol of sodium lauryl sulphate (C_12_H_25_NaSO_4_) as a surfactant (Sigma, 99%) and 8 mL of C_6_H_14_ as solvent. The resulting mixture was then stirred for 1 h at 85 °C. The resulting solution was placed in the ultrasonic device and exposed to ultrasonic irradiation under optimal conditions, which included an ultrasonic duration of 21 min, a power of 175 W, and an ultrasonic temperature of 40 °C. Cu-MOF crystals form after 30 min and are separated by centrifugation and washed with DMF.

### Characterization

The X-ray diffraction (XRD) employed for characterization and determination of the crystalline structure and phases during the synthesis of Cu-MOF. To achieve this aim, a powder X-ray diffractometer (Expert MPD, pananalytical, CuKα = 0.154.6 nm) were used in the range of 2θ = 4 – 30 degree with the step width of 0.05 degree. Scanning electron microscope (SEM, model Em 3200, china KyKy corporation) utilized for investigation of the surface morphology. Fourier transform infrared (FT-IR; SHIMADZU FT8400 spectrometer) with a Bruker- tensor 27 series was utilized for determination of vibrational frequency of the prepared samples in the range of 500 and 4000 cm^−1^. Porosities, surface area and pore textural characteristics of samples were determined by adsorption/desorption measures (BET, Belsorp mini II) at 77 K in N_2_ atmosphere. The absorbance was read by spectrophotometer (BioTek Instruments, Inc., Bad Friedrichshall, Germany).

### Anticancer activities

Anticancer activity of Cu-MOF was evaluated using MCF-7 breast cancer cells and the MTT cell viability assay according previously reported methods [26, 27]. MCF-7 cells were isolated from the pleural effusion of a 58-year-old woman with metastatic disease. In pellet culture system consisted of control medium including RPMI 1640, 10% FBS and 100 µL of penicillin G/streptomycin mixtures, cells were cultured for a period of 2 weeks. During cell culture, cell passaging was performed by trypsinization and washing was by phosphate-buffered saline. The cells with density of 1.2 × 10^4^ (cells/well) were seeded in 96-well plates and for 24 h incubated in condition of 37 °C and 5% CO_2_. With concentrations of 5, 10, 20, 40, 80, 120 and 200 μg/mL of Cu-MOF, cells were treated for 24 and 48 h. Then, the medium was then removed and 50 µL/well of MTT solutions (2 mg/mL in PBS) and 150 µL/well of fresh medium were added and incubated for 4 h. Finally, after removing the MTT solutions, to solubilize the formazan crystals, 200 μl of DMSO was added and at 570 nm, the absorbance was read using a spectrophotometer.

## Results and discussion

**Fig. 1:**
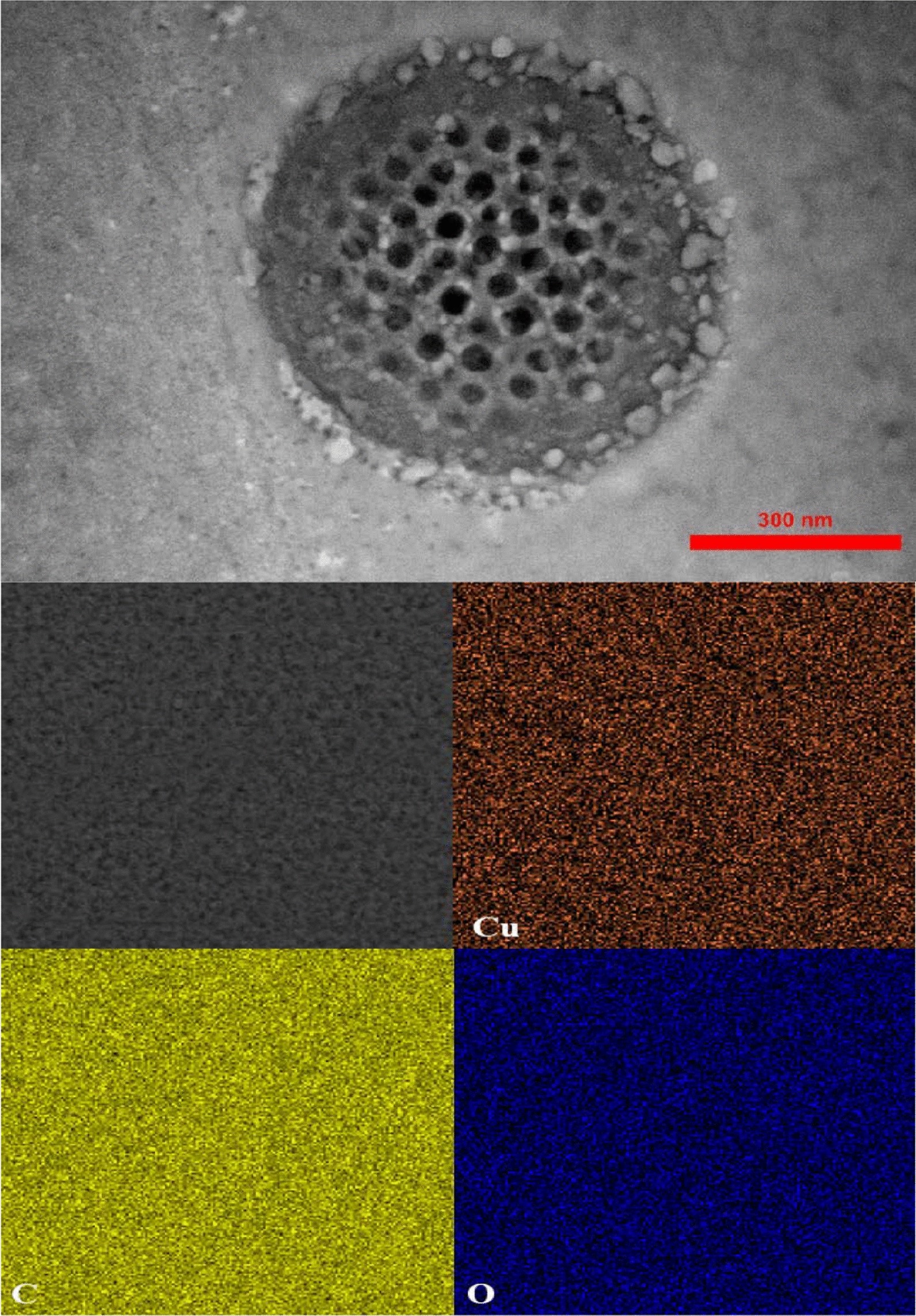
TEM image of the Cu-MOF and its mapping

**Fig. 2:**
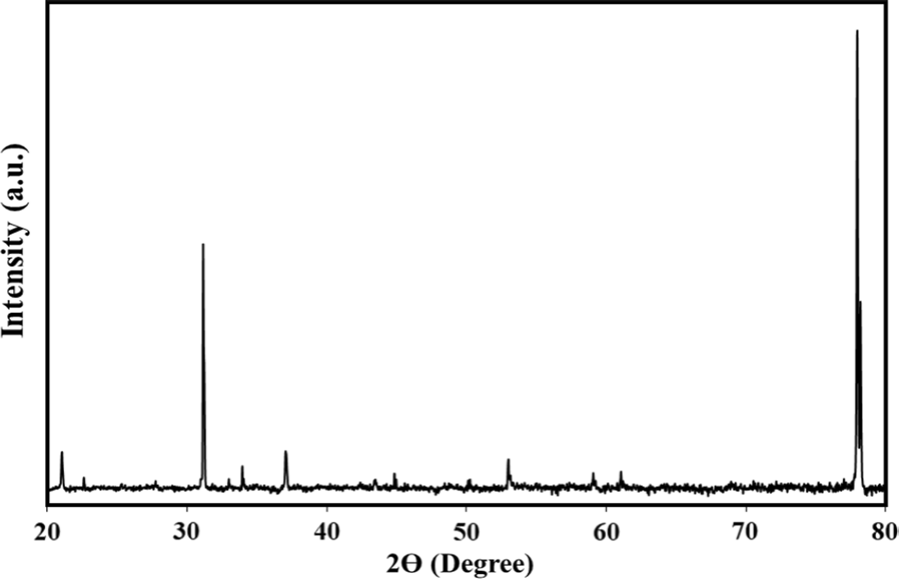
The X-ray diffraction pattern of Cu-MOF

TEM image was used to investigate the topographical study of Cu-MOF, as shown in Fig. 1. It is clear that the morphology of Cu-MOF was porous and spherical and the average particle size distribution is about 50 nm. As a result, the anticancer properties of the sample were investigated. Figure 2 depicts the phase formation and purity of the samples as determined by X-ray diffraction. The sample pattern belongs to Cu-MOF, the peaks marked with a circle at 31°, 37°,43°, 52°, 59°, and 77° for Cu-MOF (JCPDS01-072–0075) [28, 29]. The crystallite size was calculated to be 45 nm using the Debye–Scherrer formula (D = 0.9 λ/β cos θ, where crystal size is expressed by D, X-ray wavelength is expressed by λ and Braggs angle in radians is given by θ is the, and full width at half maximum (FWHM) of the peak in radians is expressed by β.

**Fig. 3:**
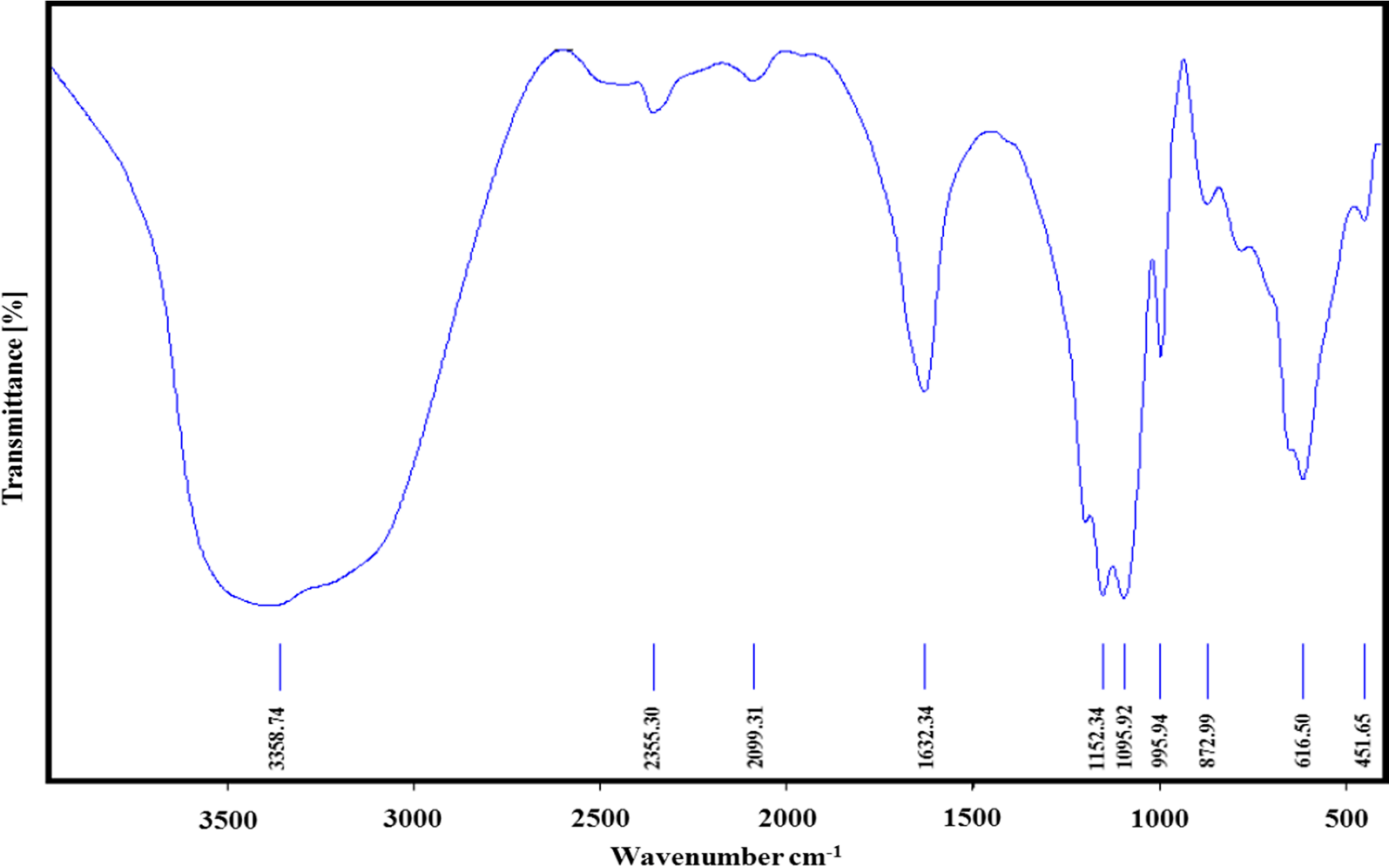
The FTIR spectrum of Cu-MOF

**Fig. 4:**
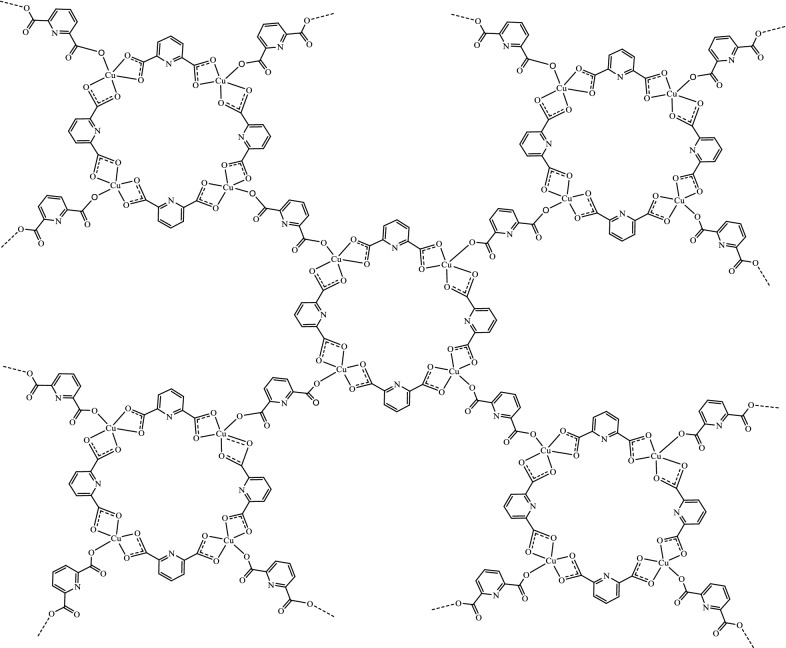
The proposed structures of Cu-MOF

The FTIR spectrum of Cu-MOF is depicted in Fig. 3. Surface water is present in the Cu-MOF structure, and as a result, the O–H broad stretching band appears at 3358 cm^−1^. [30, 31] The weak bands at 2355 and 2099 cm^−1^ are attributed to COO stretching vibrations present in 2,6-pyridine dicarboxylic acid which serves as organic linker present in Cu-MOF. The bands appeared at 1632 cm^−1^ is attributed C = O stretching vibrations [16]. The strong absorption bands at 1095 and 1152 corresponds to the asymmetric and symmetric C-O stretching [32, 33]. Absorption bands at 872 and 995 cm^−1^ are attributed to C-H symmetric and asymmetric stretching vibrations [34]. The peaks observed between 451 and 616 cm^−1^ are attributed to Cu–O stretching in Cu-MOF [35, 36]. According to the FTIR spectrum, the final structures of Cu-MOF nanostructures are suggested in Fig. 4.

**Fig. 5:**
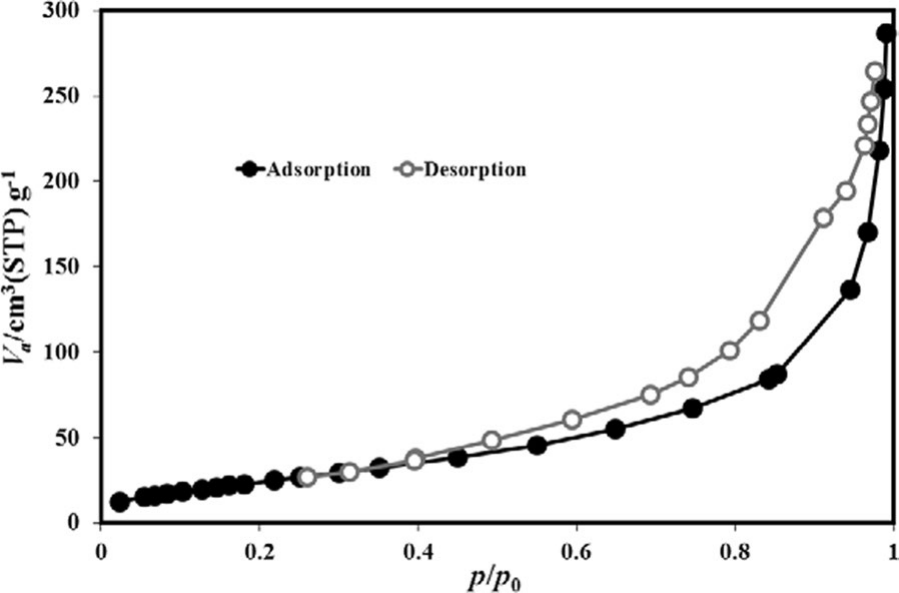
Nitrogen adsorption desorption curve for Cu-MOF at 77 K

The nitrogen adsorption/desorption results of Cu-MOF structures at 77 K are shown in Fig. 5 It (Fig. 5) depicts isotherms of the type (I) in the IUPAC classification, which is an example of microporous materials. [37] The early isotherm's dramatic increase and high N_2_ uptake indicate a high proportion of microporous. Furthermore, the amount of microporous is very low because the samples' isotherms in the high pressure region show no obvious hysteresis and tail. [38] In addition, the calculated N_2_ adsorption/desorption isotherms were related to textural parameters. The surface area of Cu-MOF measured using BET analysis was 284.94 m^2^/g. As a result, the high surface area and porosity of this sample are determined by N_2_ uptake by Cu-MOF.

**Fig. 6:**
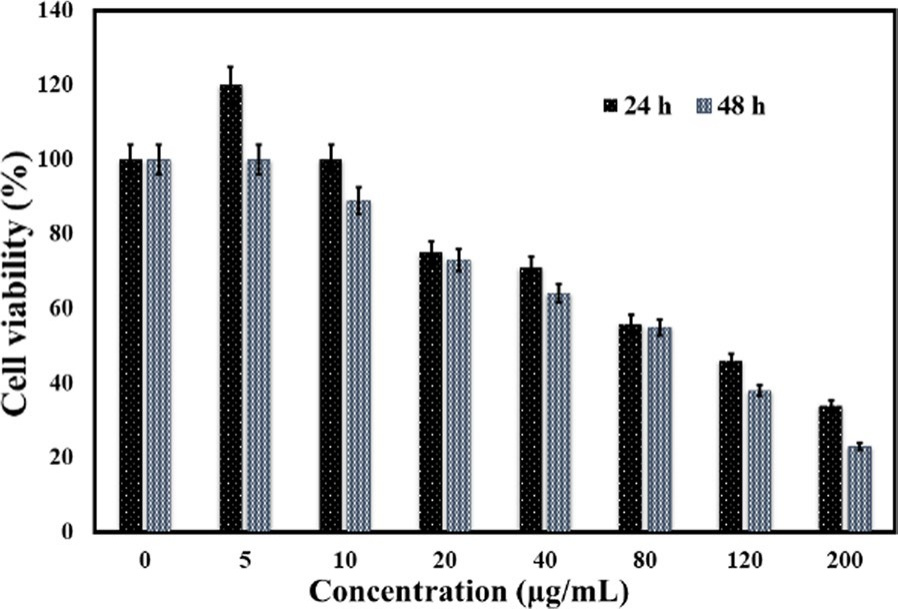
Anticancer results of Cu-MOF in MCF-7 breast cancer cell for 24 and 48 h. Data represents mean (n = 3) ± SD

Cu-MOF cytotoxicity was tested on MCF-7 breast cancer cells and the results are shown in Fig. 6. Based on the results of Fig. 6, after 48 h with high concentrations of Cu-MOF (200 g/mL), cell proliferation and viability were observed to be approximately 24% higher than the control. The IC_50_ value for exposure of Cu-MOF at 24 h, 131 μg/mL was obtained and following at 48 h, 109 μg/mL was obtained. Therefore, breast cancer cell survival was dependent on concentrations of Cu-MOF and time of incubation.

## Conclusion

Cu-MOF was prepared using UARM method and it was further characterized by FT-IR, XRD, SEM and nitrogen adsorption/desorption analysis. It has shown reasonably good anticancer activities against MCF-7 breast cancer cells. In summary, the synthesized Cu-MOF exhibited anti-cancer properties against MCF-7 breast cancer with an IC_50_ value of 109 μg/mL in 48 h and viability about 24% with the highest test concentration (200 μg/mL) was obtained at 48 h.

## Data Availability

The datasets used and/or analyzed during the current study available from the corresponding author on request.
